# The prevalence of post-traumatic stress disorder in Syrian refugees increased after long-distance migration

**DOI:** 10.1192/j.eurpsy.2022.563

**Published:** 2022-09-01

**Authors:** A.H. Eiset, M. Aoun, M. Stougaard, A. Gottlieb, R. Haddad, M. Frydenberg, W. Naja

**Affiliations:** 1 Aarhus University Hospital-Psychiatry, Depatrment Of Affective Disorders, Aarhus N, Denmark; 2 Lebanese University, Faculty Of Medical Sciences, Beirut, Lebanon; 3 Consultant, Biostatistician, Trige, Denmark; 4 King Hussein Cancer Center, King Hussein Cancer Center, Amman, Jordan

**Keywords:** Refugees, cross-sectional, Human migration, Post-traumatic stress disorder

## Abstract

**Introduction:**

Refugees are forced migrants but there is a large variation in the distance that refugees cover and there is a knowledge gap on how this may affect refugees’ health and health care needs.

**Objectives:**

Herein, we investigate the association between long-distance migration and post-traumatic stress disorder (PTSD), a serious psychiatric disorder associated with deteriorating mental and somatic health and highly prevalent in refugees.

**Methods:**

Included were 712 adult Syrian refugees and asylum seekers in Lebanon and Denmark arriving no more than 12 months prior to inclusion. The Harvard Trauma Questionnaire was used to assess PTSD and the estimate of association was obtained by multiply imputing missing data and adjusting for confounding by propensity score-weighting with covariates age, sex, socioeconomic status, trauma experience, and WHO-5-score, reporting the bootstrap 95-percentile confidence interval (95% CI). Additionally, a number of sensitivity analysis were carried out.

**Results:**

The prevalence of PTSD was high in both Lebanon (55%) and Denmark (60%) and long-distance migration was associated with a 9 percentage point (95% CI [-1; 19]) increase in the prevalence of PTSD among newly arrived Syrian refugees and asylum-seekers.

**Conclusions:**

In the present study the prevalence of PTSD increased after long-distance migration which may support considering “long-distance migration” in refugee health screenings and in particular when assessing the risk of post-traumatic stress disorder. This is a first step in examining the health effects of migration on refugee health.

**Disclosure:**

No significant relationships.

